# Core features and inherent diversity of post-acute infection syndromes

**DOI:** 10.3389/fimmu.2025.1509131

**Published:** 2025-06-03

**Authors:** Alain Trautmann

**Affiliations:** Institut Cochin, Inserm U1016, CNRS UMR8104, Université Paris-Cité, Paris, France

**Keywords:** post-acute infection syndromes, long Covid, ME/CFS, inflammation, stress, dysautonomia, hypothalamus-pituitary-adrenal axis (HPA axis), microbiota-gut-brain axis

## Abstract

Post-acute infection syndromes (PAIS), i.e., long-lasting pathologies subsequent to infections that do not properly resolve, have both a common core and a broad diversity of manifestations. PAIS include a group of core symptoms (pathological fatigue, cognitive problems, sleep disorders and pain) accompanied by a large set of diverse symptoms. Core and diverse additional symptoms, which can persist for years, exhibiting periods of relapses and remissions, usually start suddenly after an apparently common infection. PAIS display highly variable clinical features depending on the nature of the initial pathogen, and to an even larger extent, on the diversity of preexisting individual terrains in which PAIS are rooted. In a first part, I discuss biological issues related to the persistence of microbial antigens, dysregulated immune responses, reactivation of latent viruses, different potential self-sustained inflammatory loops, mitochondrial dysfunction, metabolic disorders in the tryptophan- kynurenin pathway (TKP) with impact on serotonin, and consequences of a dysfunctional bidirectional microbiota-gut-brain axis. The second part deals with the nervous system dependence of PAIS. I rely on the concept of interoception, the process by which the brain senses, integrates and interprets signals originating from within the body, and sends feebacks aimed at maintaining homeostasis. Interoception is central for understanding the origin of fatigue, dysautonomia, dysfunctioning of the hypothalamus-pituitary-adrenal (HPA) axis, and its relation with stress, inflammation or depression. I propose that all individual predispositions leading to self-sustained vicious circles constitute building blocks that can self-assemble in many possible ways, to give rise to both core and diverse features of PAIS. A useful discrimination between different PAIS subtypes should be obtained with a composite profiling including biomarkers, questionnaires and functional tests so as to take into account PAIS multidimensionality.

## Introduction

In most cases, an acute common infection lasting a few days is followed by the progressive resolution of this infection, with no persisting symptoms, even though the immune system keeps a memory of the infectious event (1). This memory may be registered both in the adaptive immune system (memory T and B cells), and in the innate immune system (trained immunity), through epigenetic modifications of innate immune cells, epithelial cells, and their corresponding stem cells. However, in a minority of patients, an infection may not properly resolve, leading to a post-acute infection syndrome (PAIS), a persistent pathology that may last for years (2–5).The review focuses on three PAIS, i.e. long COVID, long Lyme/PTLDS (post-treatment Lyme disease syndrome) and myalgic encephalomyelitis/chronic fatigue syndrome (ME/CFS).

If long COVID is, by definition, induced by SARS-CoV-2 and long Lyme by *Borrelia*, the case of ME/CFS is far less clear. Its core symptoms (extenuating fatigue, post exertional exacerbation, cognitive dysfunction, sleep disorders and pain) are shared with the other PAIS (6–8), but no unique, identified pathogen appears responsible for its triggering. In most cases, its post-infectious origin appears highly likely in view of sudden epidemics of ME/CFS, which have been listed by Warren Tate and colleagues (8), and have been observed in Los Angeles (1934), in Iceland (1946–1948), at the Royal Free Hospital in London (1955), at the Incline Village, in Nevada (1984), and in Tapanui, New Zealand (1984). In each case, no pathogen was clearly identified. EBV (Epstein-Barr virus) appears as a likely and frequent candidate for ME/CFS triggering (9, 10), but it is not the only possible ME/CFS inducer. The great difficulty in diagnosing ME/CFS has led to many successive definitions of this disease. Rather than a distinct, well-defined PAIS, ME/CFS may be viewed as an ensemble of PAIS that could be triggered by different pathogens, and that may include severe forms of long COVID and long Lyme. Thus, the frontiers between between ME/CFS and other PAIS are far from being sharp. However, in what follows, and in particular in **Table 1**, which will be described later, the term ME/CFS will point to long diseases in which the triggering pathogen is neither SARS-CoV-2 nor *Borrelia* (otherwise, they would be called long COVID or long Lyme). The heterogeneous nature of this ensemble could have contributed to the poor recognition of the severity of the disease. For ME/CFS recognition, long COVID will be a game changer and, in return, the abundance of data and analyses concerning ME/CFS could help the understanding of mechanisms underlying other PAIS.

**Table 1:** Core and diverse PAIS symptoms.

Symptoms	Long COVID	ME/CFS	Long Lyme/PTLDS
Core symptoms			
Fatigue	++	++	++
Brain fog (impaired memory, impaired attention)	++	++	++
Myalgia	++	++	++
Unrefreshing sleep	++	++	++
Headaches	++	++	++
Diverse symptoms			
Cardiothoracic symptoms			
Palpitations	++	++	++
Post-exertional malaise (PEM) (exhaustion after physical or mental exercise)	++	++	p
Shortness of breath	++	p	+
Orthostatic intolerance (POTS)	++	+	p
Cough	+	–	p
Myocarditis, pericarditis	p	–	+
Neurologic and neurocognitive symptoms			
Vertigo or ataxia	++	++	+
Hot and cold spells	+	++	+
Radiculitis	+	–	++
Paresthesia, tinnitus, sensitive disturbances	++	+	++
Facial paralysis	–	–	+
Motor disturbances	+		p
Muscle and joint symptoms			
Joint pain, arthritis	+	++	++
Muscle weakness	+	++	+
Digestive symptoms			
Nausea	++	++	+
Diarrhea or constipation	+	++	+
Epigastralgia, reflux	+		p
Intestinal pain	+	p	+
Poor appetite	+	p	+
Bloating	+	p	+
Ear noose throat symptoms			
Aching throat	+	+	+
Decreased smell and taste	++	–	–
Hypersensitivity to noise, odors	+	+	+
Ophtalmologic symptoms			
Blurred vision	+	+	+
Eye dryness	++		++
Chronic ear pain or otitis	+		+
Hypersensitivity to light	+	+	+
Cutaneous symptoms			
Cutaneous lesions such as rash, eczema, urticaria	+	p	p
Hair loss	+		++
Spontaneous cutaneous hematomas	+		+
Urino genitary symptoms			
Urinary impairments	+	+	p
Menstruation and libido disorders	+	p	+
Psychological and psychiatric symptoms			
Increased emotionality and irritability	++	p	++
Secondary anxiety	++	+	+
Secondary depression	+	+	+
Other			
Profuse sweats	+	+	+
Hypersensitive to sound, smells, food and medicines	++	+	++
Painful lymph nodes	–	+	p
Mild fever	+	p	+
Flu-like symptoms	+	+	+
Recurrent infections	+	+	p
Gain or loss of weight	++	p	+
Reactivation of autoimmune disorders such as thyroiditis	+		+

++ Very frequent (>50%).

+ Frequent. (20-50%).

p Present, but at an unknown frequency.

- Absent.

Empty The information is lacking.

All PAIS display a striking heterogeneity. Indeed, for each PAIS, the number of symptoms, as well as their severity, vary greatly depending on individuals. Heterogeneity intrinsic to each PAIS represents a major obstacle to the design and implementation of clinical trials. To be able to properly manage PAIS, it is necessary to address this heterogeneity and, first and foremost, to try unravel its origin, rooted in a bidirectional interaction between the brain and the others parts of the body. This review aims at providing a broad view of PAIS, ranging from the molecular/cellular level to the whole organism, to provide a theoretical background and to introduce notions that could help understanding how PAIS can have both clear core features and a large set of diverse symptoms. These issues will be addressed at two levels. Part I will deal with topics related to immunity, inflammation, microbial persistence, mitochondrial dysfunction and metabolism, Part II with the involvement of the nervous system in PAIS, and in a dysfunctional interoception. The microbiota-gut-brain axis issue will make the transition between the two parts.

## Part I. Immunity/inflammation and metabolism in PAIS

### Most symptoms of a PAIS are shared with other PAIS

**Table 1** summarizes the symptoms most frequently reported in long COVID, ME/CFS and long Lyme/PTLDS. One can see that, with variable frequencies, most symptoms are shared by the different PAIS, and that there are very few disease-specific features. For instance, SARS-CoV-2 is a virus with a respiratory and ear nose throat transmission, which affects the olfactory mucosa and has a facilitated neuroinvasion potential (11–16). In addition, it has a strong tropism for blood vessels and digestive tissue due to its specific binding to the ACE2 receptor, well expressed in those tissues. In line with these specific features, a number of long COVID symptoms (eg, anosmia, frequent dyspnea, vascular and coagulation issues) are not shared by other PAIS (6, 7). As for *Borrelia*-induced PAIS, i.e., long Lyme/PTLDS, the tropism of *Borrelia* for connective tissues likely accounts for the frequent occurrence of Lyme-associated arthritis, myalgia and paresthesia (8). In long Lyme, persistent disorders and symptoms associated with this tropism could be due to local bacterial persistence, and/or to self-sustained local disorders initially triggered by *Borrelia*.

This table was constructed on the basis of informations provided by patients and doctors who are expert in one of these diseases, and on published papers, in particular (6–8, 17–25).

### PAIS triggering and perpetuation

In this review, I propose that PAIS should be regarded as a set of pathologies affecting the proper *resolution* of infection-induced inflammatory and neuro-immune responses. I make the hypothesis that the susceptibility to PAIS depends on each individual terrain, including genetics, epigenetics, lifestyle, infectious history, and so on. The infectious agent would trigger a PAIS only when there is an appropriate preexisting individual terrain, which is highly diverse. It is this diversity which would explain the great heterogeneity of PAIS manifestations. These individual susceptibilities may have gone unnoticed before the deleterious triggering infection, except for discreet warning signs. For instance, in patients suffering from ME/CFS or long COVID, childhood infectious illnesses have been reported as abnormally frequent, symptomatic and long to heal (up to 2 months) (26).

As will be examined in detail in this review, a whole range of different susceptibilities may facilitate the development of a PAIS. For instance, a propensity to make an excessively intense innate immune response, or on the contrary the unability to control an infection, the pathogen persistence (potentially due to the previous cause), or a suboptimal functioning of mitochondria, leading to an excess production of reactive oxygen species (ROS), or a suboptimal functioning of the neurovegetative nervous system, facilitating the triggering of dysautonomia, or a propensity do develop autoimmune diseases. It will be shown that these various and non excusive susceptibilities may lead to a set of positive feedback loops that can become self-sustained, even in the absence of a persistent pathogen, but more efficiently in case of pathogen persistence. It will also be proposed that several of these different vicious circles may self-assemble in ways that facilitate disease perpetuation.

The diversity of the terrain weaknesses at the origin of PAIS development, and the perpetuation mechanisms that are installed are likely to explain the diversity of long-term evolution of these diseases. In one study, after one year of long COVID, no improvement was reported by 63.7% of the patients (25). In another report, after a two-year follow-up of long COVID patients, 91% of them were slowly improving with time, 5% rapidly improving, and 4% of patients remained in a severe stable state (27). According to a careful longitudinal study on the last 6 months period (28), only 2% of ME/CFS patients considered that their health was improving. For the vast majority (68%) of the patients, the disease fluctuates, in a relapsing-remitting fashion, whereas for 30% of the patients, the disease either slowly worsens over time or is persistent, but never improves. Thus, for 98% of the ME/CFS patients, this disease appears very long-term or permanent.

Despite the fact that the existence of flares and remissions is a common and important feature of PAIS, the oscillating systems underlying these flares are far from being understood. Note however that a clever model of flares and remissions in multiple sclerosis (MS) has been proposed by the team of Uri Alon (29). In this excitable model of the immune system, with stochastic flares, a key role is given to the interaction between auto-reactive T cells and regulatory T cells (T_Reg_). The model describes a positive feedback (more cytotoxicity releases more auto-antigens) and a negative one (cytotoxicity stimulates immunoregulatory T_Reg_). Several experimental observations on flares and remissions in MS were correctly predicted by this model. It has also been proposed that flares in multiple sclerosis could correspond to phases of reactivation of the latent herpesvirus HHV-6 (30). Given the frequent occurrence of reactivated latent viruses in PAIS (see later), the generality of such a mechanism deserves to be examined. I suggest that other possible flares inducers could be infections by other pathogens, or excessive cognitive or physical efforts. The discovery of other flare inducers and alternative oscillating models is eagerly awaited.

### PAIS may be induced by many different pathogens

PAIS may be induced by a broad array of pathogens. As shown in **Table 2**, most PAIS-inducing pathogens are RNA viruses, but many RNA viruses, like measles virus, do not trigger PAIS. Additional PAIS-inducing pathogens include one DNA virus, EBV, and a bacteria (*Borrelia*).

**Table 2:** PAIS may be triggered by a variety of pathogens.

Pathogens	Disease names	Sites of pathogen persistence	References
Viruses			
SARS-CoV-2	Long COVID, PASC	Lung, brain, gut, heart, lymphoid tissue, ear nose throat tissue.	(2, 31–37)
SARS	SARS		(38, 39)
Ebola	post-Ebola syndrome	Testes, eye, brain	(40–43)
Dengue		brain	(44, 45)
Polio	Post-polio Syndrome	Brain, spinal chord	(46–48)
Other enteroviruses	ME/CFS Viral heart disease	Stomach, heart	(49–51)
Chikungunya	Chikungunya chronic disease	Joints	(52–54)
West Nile virus		Kidney, brain	(55–57)
H1N1 influenza	ME/CFS	No evidence	(58)
EBV	ME/CFS	B cells	(9, 10)
Non viral pathogen			
*Borrelia*	Post-treatment Lyme Disease	Brain, synovial fluid	(59–63)

### Pathogen persistence and reactivation of latent viruses in PAIS

A major possible cause of PAIS resides in the non-eradication and persistence of the pathogen, which could sustain a long lasting inflammatory/immune response (64). Signs of viral persistence have been reported in different tissues and cells of long COVID patients, particularly in the gut (32, 34, 65, 66), brain or cardiac tissue (36), monocytes (67), and in megacaryocytes and platelets (68). Platelets could well be transient carriers of the virus and megacaryocytes the corresponding virus reservoirs, as it has been shown for HIV (69). In animal models of COVID, virus persistence as also been demonstrated in the central nervous system (12) and in pulmonary tissues (70). Diane Griffin has recently established a comprehensive summary of the different viruses able to lead to RNA persistence and to PAIS, of the location of RNA reservoirs (mostly intracellular), of affected organs, and of clinical consequences associated with this persistence. Notably, even in the absence of viral replication, the mere persistence of intracellular viral RNA may be sufficient to chronicize an innate immune response (71). Numerous viruses have a demonstrable potential for persistence. This is the case not only for SARS-CoV-2, but also for herpesviruses (EBV, CMV, HHV-6) (72), for EBOLA (40, 41), for enteroviruses (73), or Chikungunya (74). In long Lyme/PTLDS, the persistence of *Borrelia*, the initiating pathogen, has also occasionally been reported (75, 76).

The elevated plama level of anti-SARS-CoV-2 IgA observed in severe long COVID could be a sign of virus-induced mucosal persistent inflammation (77, 78). An elevated level of IFN-α in the blood may also be evocative of virus persistence. For comparison, untreated HIV patients have an elevated level of IFN-α, which rapidly drops following tritherapy (79). However, an elevated IFN-α is not necessarily virus-induced, as it has also been reported in *Borrelia*-induced long Lyme/PTLDS (80, 81).

To the possible persistence of the initial pathogen that triggered the PAIS, one should add the reactivation of latent viruses such as EBV, HHV-6 or VZV (82–85), all frequently observed in PAIS, possibly as a result of a reduced efficiency of their control by the immune system. It is plausible that this reactivation may further contribute to the disease chronicity. Moreover, additional pathogens may emerge, following the translocation of gut bacteria in the blood through an inflamed, damaged gut mucosa (as discussed later). In all these cases, the pathogens to be neutralized are not only the initial one but also its followers. Similarly, increased transcription of human endogenous retroviruses (HERV) has been reported in several PAIS (86), and in MS. This has led to an encouraging clinical trial for the treatment of MS with temelimab, an anti-HERV monoclonal antibody (87). However, a recent trial with temelimab has been unsuccessful in long COVID.^1^

Taken together, these data show that PAIS may be induced by a large set of diverse pathogens, and different phenomena may contribute to the disease severity. They include namely pathogen persistence, reactivation of latent viruses, and a series of self-sustained loops and cascades of events, which will be discussed below.

## Mechanisms underlying the diversity in the clinical presentation of PAIS

### Poorly fitted immune responses

There is an increased risk of developing long COVID for patients who suffered from a very symptomatic form of COVID-19 and have been hospitalized (88–90). Some of these severe forms appear associated with insufficient innate immune responses, involving namely IFN-α (91, 92). However, even though symptoms associated with the acute phase of COVID are predictors of the probability of developing long COVID (91–94), a number of cases of long COVID have been observed after moderate initial COVID (95).

For eliminating the virus, an efficient antibody response, properly supported by CD4^+^ T cells appears required (96). However, long COVID patients often have high level of anti-SARS-CoV-2 antibodies, which shows that a good antibody response may not be sufficient (97). An insufficient antiviral antibody esponse may also be problematic, as shown by the recent observation that two subgroups may be distinguished in long COVID: some patients are seropositive (with the presence of serum antibodies directed against multiple SARS-CoV-2 proteins), while others remain seronegative (98). Seronegative patients also had fewer SARS-CoV-2-specific CD4 T cells than seropositive patients or controls; their global adaptive immune response was ineffective.

It appears that a well fitted immune response is required for preventing PAIS development. Stimulating hypotheses may be drawn from the observation that bats can be persistently infected with many viruses without showing clinical symptoms (99). Bats are equipped with an apparently optimized anti-viral IFN-α response, in terms of amplitude (neither too weak nor oversized) and kinetics (preexisting rather than delayed) (100, 101). Note that, while the level of IFN-α in human serum is almost nil in the absence of infection, there is a low but detectable level of IFN-α in bat serum (102, 103). The existence of a weak baseline IFN-α-dependent signal (104) leading to an *inflammatory tone* (105) seems essential to enable an effective anti-infectious response, just like engine warm up right before a car race. Indeed, as a general rule, the absence of an inflammatory tone leads to a blunted efficacy of the immune response (106). These features contribute to the exceptional resistance of bats to viral infections, together with other features such as a better resistance to oxidative stress (107). In a mirror view, one can speculate that a poorly fitted immune response, either too weak to control the pathogen, or associated with an excessive inflammation, may contribute to the triggering of PAIS.

Murine studies have demonstrated the existence of a link between vigorous anti-infectious responses and propensity to develop autoimmune diseases (108). The same association may be observed in women (109). The anti-infectious response of women is usually more vigorous than that of men. One of the reasons why acute COVID has killed more men than women could lie in the fact that the IFN-α response to a viral infection is stronger in women than in men (110, 111). This fact is likely linked to the influence of oestrogens and to the fact that a large part of the immunity genes are located on the X chromosome (112). Thus, compared to men, women have a better ability to fight an infection but a poorer ability to resolve infection-induced immune responses.

### Autoimmunity

Most autoimmune diseases (113–115), as well as PAIS, appear after infections from which a majority of people recover without sequaele. This suggests that autoimmune diseases and PAIS could possibly share common predisposing terrains. They correspond to distinct but related diseases, given that autoimmunity is by definition the dominant problem in one case, and not in the other, even if autoreactivity may play a role in PAIS. A key link between infection and autoreactivity is the existence of pre-existing quiescent auto-reactive T cells, which may be initially activated by their cytokine receptors to IL-2 or IL-15, during an anti-infectious immune response. These bystander auto-reactive T cells are then amplified upon recognition of self antigens (116, 117).

There is no shared signature of autoreactivity that would be specific for long COVID patients (118), and the functional importance of autoimmunity in PAIS such as long COVID, ME/CFS or long Lyme/PTLDS remains to be established. However, signs of autoimmunity have been reported in the different PAIS. Thus, anti-neural antibody reactivity was found to be significantly higher in the long Lyme/PTLDS group than in the post-Lyme healthy one (119). Autoantibodies against G-protein coupled receptors are found in ME/CFS patients and in patients with persistent long-COVID-19 symptoms (120, 121). Even though some new autoantibodies are shared in post-COVID people with or without persisting symptoms (118), persistently positive anti-nuclear autoantibodies at 12 months post-COVID are associated with persisting symptoms and inflammation in a subset of long COVID patients (122), and several peripheral nervous system antibodies demonstrate statistically significant differences depending on severity (123). An autoimmune reactivity against tight junctions (zonulin and occludin) and neuronal antigens has also been reported in long COVID. Note that in addition to canonical (barrier-related) functions, zonulin and occludin have other key, noncanonical functions that allow them namely to regulate epithelial apoptosis and proliferation and to facilitate viral entry (124).

Finally, a possible contribution of coronavirus-induced autoantibodies to long COVID has been recently suggested by the demonstration that transfer of IgG from long COVID patients to mice replicates some neurological symptoms, pointing to a causative role of IgG in long COVID pathogenesis (125, 126). The question of toxic IgG remains a potentially important but yet unsolved issue, which deserves to be examined in the different PAIS.

Taken together, these findings underline the existence shared features between (post-infectious) autoimmune diseases and post-acute infection syndromes.

### PAIS and inflammation

The importance of inflammation in PAIS remains a disputed issue for several reasons. One is linked to the objective diversity of inflammatory signs. Depending on the PAIS severity and on the individual, standard inflammatory tests such as CRP, may be completely normal or slightly elevated. For instance, for long COVID, CRP level is normal in moderate forms, and frequently positive in severe forms (127–129). Note however that a well resolved viral infection, without associated persisting symptoms, also creates an immunological scar with persisting immune activation and increased cytokine production several months after symptom resolution (130, 131). Biomarkers associated with long COVID thus have to be compared to biomarkers associated with properly resolved COVID.

The link between PAIS and inflammation has been mostly documented for long COVID, as will be seen below. However, neuroinflammation has also been evidenced by ^11^C-(R)-PK11195 PET imaging to be associated with ME/CFS (132), and shown to contribute to the pathophysiology of this disease (133, 134). As for neuroborreliosis, it is associated with the elevation of inflammatory cytokines in the CSF, like IFN-γ (135) or TNF-α (136). Such inflammatory cytokines may either cross the blood-brain-barrier, especially if it is damaged, or be produced *in situ* by reactive astrocytes and microglia (137). Moreover, long Lyme patients are familiar with the Jarisch-Herxheimer Reaction, a potentially intense inflammatory response, which can be elicited by an antibiotic treatment of a persistent borreliosis (138).

Several teams, using different sets of biomarkers, have distinguished inflammatory and non-inflammatory subsets of long COVID patients (129, 131). There is so far no inflammatory marker that has been systematically found in PAIS, but it should be remembered that a persistent inflammation affecting an organ, e.g., neuroinflammation, may remain local and may only minimally be reflected in blood biomarkers. However, inflammation-related vascular problems have been frequently reported. Thus, elevated levels of some blood biomarkers such as VEGF underline the frequent occurrence, in long COVID, of endothelial vasculitis (139, 140), dysregulated blood coagulation, with fibrin amyloid microclots and platelet pathology (141), vascular damage, repair and remodeling (142), thromboinflammation and dysregulation of the complement cascade (143).

Let me now examine how the dysfunctioning of different cells of the immune system may contribute to long COVID and other PAIS.

### Neutrophils and platelets

Neutrophils are key cells involved not only in acute but also in chronic inflammation. In the event of infection, neutrophils are swiftly activated and produce ROS, which may contribute to decondensing mitochondrial and cellular DNA. The expulsed chromatin, coated with antimicrobial proteins, forms NETs (neutrophil extracellular traps), able not only to trap pathogenic microorganisms, but also to propagate inflammation and to favour the induction of microclots following platelet binding (144, 145). NETs have been shown to be involved in acute (146) and long COVID (147). In long COVID, activated platelets, monocytes and endothelial cells interact to create a prothrombotic environment and induce vasculitis (141, 148–150). In ME/CFS patients, microclots (151), as well as an improper platelet activation after a physical effort (152) have also been reported.

### Mast cells, histamine and PAIS

Mast cells, innate immune cells found in connective tissues throughout the body, are most prevalent at tissue-environment interfaces and perivascularly. This allows them to play a key role in fast anti-infectious inflammatory responses. They possess multiple cell-surface receptors which react to various stimuli and, after activation, release numerous mediators including histamine, heparin, cytokines, prostaglandins, leukotrienes and proteases (153). However, their contribution to inflammation may sometimes become excessive, for instance in allergy.

The fact that an atopic terrain is a risk factor for long COVID (154, 155), and that mast cell activation symptoms are frequently present in long COVID, has led to the use of anti-histaminic treatments which have proven their efficacy, for some patients, on several long COVID symptoms, including fatigue, digestive and neurocognitive symptoms (156–158) (and Dominique Salmon, personal communication). It has been proposed that COVID-19 infection could lead to the activation of normal mast cells by persistent viral particles or spike proteins, or to exaggeration of a preexisting but undiagnosed mast cell activation syndrome (159, 160). No study to date has examined the presence of anomalous mast cell activation in patients with long Lyme/PTLDS (17).

### T cells and the T cell, B cell and monocyte triangle

The CD8 T cells of patients suffering form ME/CFS or long COVID have an exhausted phenotype (161–163). Patients suffering from long COVID harbored increased frequencies of CD4+ T cells with homing receptors adressing to inflamed tissues, and a Th2 bias (163). A key implication of T cells in long COVID, compatible with virus persistence, could be demonstrated by whole-body positron emission tomography imaging with a selective tracer that allows for anatomical quantitation of activated T lymphocytes (34). In numerous parts of the body, tracer uptake (and therefore T cell activation) was higher in the postacute COVID-19 group (with or without continuing symptoms) than in prepandemic controls. Moreover, T cell activation in the spinal cord and gut wall was associated with the presence of long COVID symptoms.

In inflammation and autoimmune processes, T cells, B cells (and the antibodies that they produce) and inflammatory monocytes/macrophages may form a cellular network acting in a pathogenic way. All three cell types can interact with the others and fuel a vicious activation circle (164). In particular, activated Th1 T cells produce IFN-γ, which can activate monocytes/macrophages, which in return may activate T and B cells via antigen presentation, whilst B cell-derived antibodies are able to activate monocytes/macrophages expressing the FcγRI receptor. In addition, activated T cells express CD40L, which can activate the CD40 receptor expressed by B cells and by monocytes/macrophages. Altogether, this **first self-sustained inflammatory loop** could have a particular importance in the brain, i.e., in neuroinflammation. Such a loop is of utmost importance in autoimmune diseases. It is not a specific feature of PAIS, but it may play an important role in PAIS, at least in some patients.

### Brain imaging of neuroinflammation or hypometabolism

Inflammatory cytokines are able to cross an intact blood-brain barrier. They can cross even more easily an inflamed, damaged one, and activate cytokine receptors on neurons (165), astrocytes (166) microglia (167), and brain mastocytes (168). Microglia are brain resident macrophages. They may function, like any other type of macrophage, either in a resting, homeostatic, anti-inflammatory mode, or, on the contray, in an activated, pro-inflammatory one (169, 170) susceptible of amplifying and prolong a peripheral inflammation-induced neuroinflammation. Importantly, microglial cells can both produce and be activated by inflammatory cytokines like IL-6, TNF-α and IL-1β (also produced by inflammatory monocytes) (171). This constitutes a **second potential self-sustained inflammatory loop**.

Brain imaging has provided key informations on PAIS-associated neuroinflammation, on long COVID-associated hypometabolism, and even on COVID-induced changes in brain structures. Neuroimaging has revealed the presence of neuroinflammation in MS, as reviewed in (172), in long Lyme (173), as shown by imaging glial activation, using [11C]DPA-713 PET, and in ME/CFS, as reviewed in (174). Subgroups of long COVID patients frequently display a correlation between inflammation biomarkers and fatigue plus cognitive impairmen (88, 127, 175). In addition, ^18^F-FDG PET imaging allowed to show the presence of extensive and durable hypometabolism in several brain regions of long COVID patients (176, 177), in particular in the pons and the right amygdala (178). Brain regions that were hypermetabolic during acute COVID turn out to become hypometabolic in long COVID (179). An analysis of changes in MRI-derived brain structure induced by long COVID has evidenced a reduction in grey matter thickness and tissue contrast in the orbitofrontal cortex and parahippocampal gyrus, associated with cognitive problems (180).

### Mitochondrial dysfunction in PAIS

As mitochondria constitute the key ATP power plant, i.e., the energy provider in our cells, the importance of their dysfunction in PAIS and other diseases characterized by a hypometabolic state and fatigue could well be crucial. Indeed, defects in mitochondrial function (deficiency in ATP production, excessive mitochondria-derived ROS production) in PBMCs of ME/CFS patients have been well characterized (181, 182). The PBMCs of ME/CFS patients have a reduced ability to elevate their respiration rate to compensate in times of physiological stress (183). In the blood of ME/CFS patients, there is an abnormally high level of FGF-21 (Fibroblast Growth Factor-21), which is not only a growth factor but also a hormone (184). FGF-21 is a powerful regulator of glucose and lipid metabolism, which is significantly increased under certain conditions, such as mitochondrial dysfunction (see (185) for a review). The plasma level of FGF-21 is also significanly higher in the plasma of long COVID patients with important cognitive problems (186). In a cohort of ME/CFS patients, the degree of mitochondrial dysfunction in neutrophils was strongly correlated with the severity of the illness (187). Admittedly, correlation does not imply causal relationship. However, such striking correlations suggest that the hypothesis of mitochondrial dysfunctioning in ME/CFS deserves deing pursued. Importantly, crucial fatigue, exercise intolerance and myalgia are not specific of ME/CFS, but are shared by patients suffering from primary mitochondrial diseases with mutations in either nuclear or mitochondrial DNA (188).

In mitochondrial dysfunctioning, a key issue is that of ROS. Indeed, ATP synthesis in mitochondria is systematically associated with ROS production and potential oxidative stress. Mitochondria have an efficient ROS buffering system including GSH, thioredoxin or Coenzyme Q10 (CoQ10) (189–191), and as long as ROS are eliminated by endogenous antioxidants, there is no oxidative stress. However, in the blood of ME/CFS patients, excessive oxidative stress response to exercise can be measured via different plasma markers (192), and in mitochondria from ME/CFS patients, defects have been detected in CoQ10 and in Complex V (aka ATP synthase) (182, 190). Compared to healthy controls (HC), ME/CFS patients display a pattern of cellular hypometabolism characteristic of impaired mitochondrial function (193). Excessive unbuffered ROS levels damage mitochondria, leading to less ATP and more ROS production, which creates a potential **third self-sustained inflammatory loop**. In addition, ROS-induced ROS release is a self-amplified phenomenon (194, 195). Cardiolipin is a particularly abundant phospholipid in the inner membrane of mitochondria and thus, an increase in anti-cardiolipin auto-antibodies may reveal damage in mitochondria membranes. Such anti-cardiolipin antibodies are increased in ME/CFS (196, 197), long COVID (147) and MS patients (198).

There is a tight link between ROS and inflammatory cytokines. Indeed, both TNF-α and IL-1β can stimulate ROS production and inhibit mitochondrial respiration, whilst increasing glycolytic activity and lactate production (199). Thus, inflammation impairs oxidative phosphorylation and impose a higher reliance on glycolysis, which is much less efficient for energy production.

The potential role of mitochondrial dysfunction in PAIS other than ME/CFS has not yet been widely explored. However, such a dysfunction has been described in metabolomic analyses of long COVID (186, 200). In long COVID patients, an excessively increased blood lactate accumulation during exercise has been reported (201). A similar finding had been previously reported in ME/CFS patients (202). Moreover, using proton magnetic resonance spectroscopic imaging with (1)H MRSI, significantly higher levels of ventricular cerebrospinal fluid lactate are found in ME/CFS patients compared to healthy controls (203). Given the vicious circle between ROS production and mitochondrial dysfunction mentioned above, it has been reasonably hypothesized that, in some individuals with pre-existing sub-optimal mitochondrial function, an infection can tip the host into a chronic and self-perpetuating metabolically imbalanced non-resolving state characterized by mitochondrial dysfunction, where ROS continually drive inflammation (204).

### PAIS metabolic disorder in the tryptophan- kynurenin pathway with impact on serotonin

**Figure 1:**
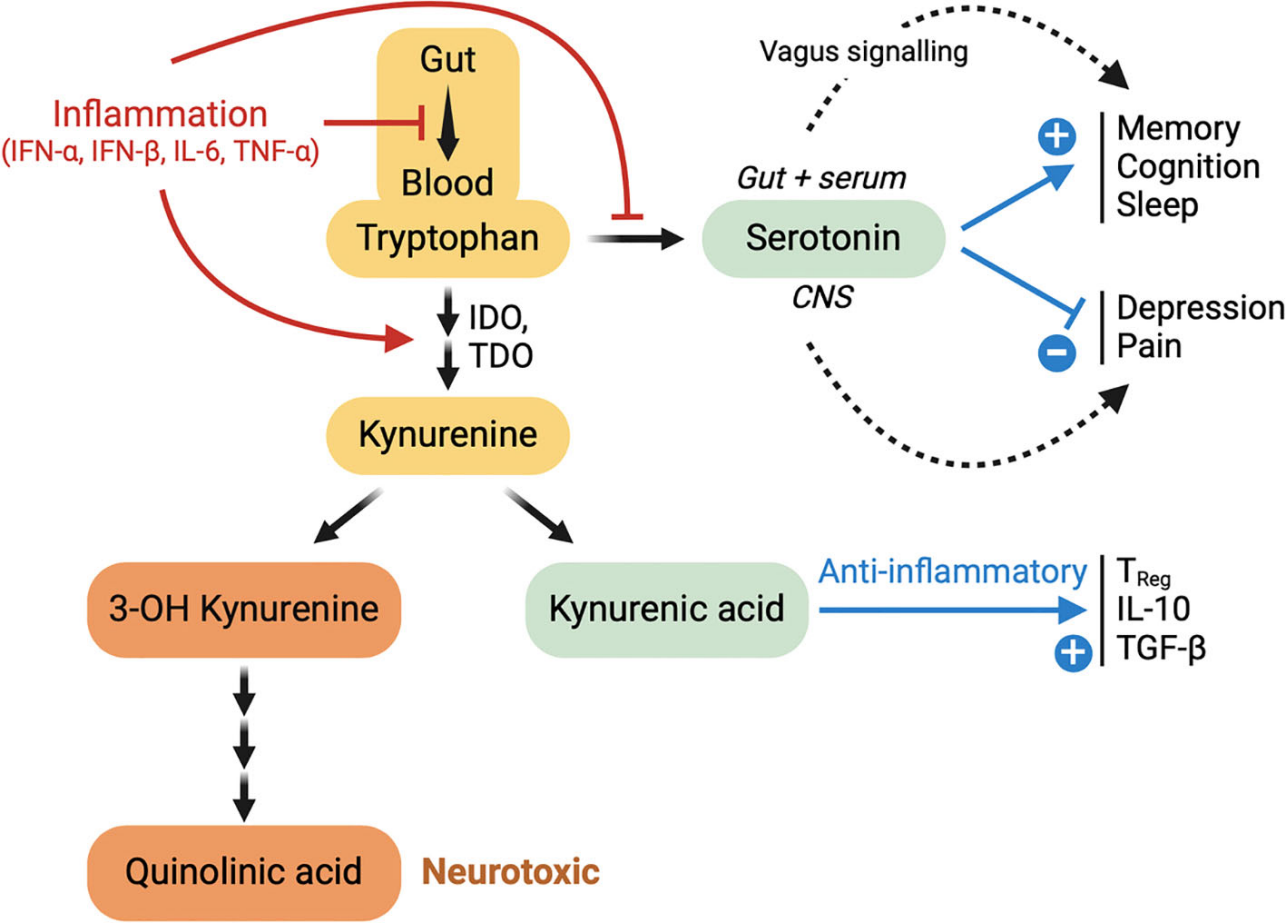
Food-derived tryptophan (trp) can be degraded in kynurenine, which can either give rise to neurotoxicity (via quinolinic acid) or to anti-inflammatory effects (via kynurenic acid). Trp is necessary to the synthesis of serotonin, both in the gut and in the brain. This synthesis is inhibited by inflammation. Gut-derived serotonin cannot cross the blood brain barrier, but may have positive effects on memory, cognition and sleep via stimulation of 5-HT3 receptors on the vagus nerve (Wong et a., 2023). Brain-derived serotonin has inhibitory effects on depression and pain.

Tryptophan (trp) is a key precursor of serotonin (5-HT), which may be produced in the gut and in the brain. In the gut, it is synthetized by enterochromatin cells, and rapidly stored in blood platelets. The high and selective expression of the 5-HT_3_ serotonin receptor on sensory neurons of the vagus nerve allow a fast central detection of peripheral serotonin changes (205). The blood-brain-barrier can be crossed by Trp, but not by serotonin (206). Therefore, cerebral serotonin is synthetized in the brain, where it plays a major role in mood regulation and, after transformation in melatonin, in sleep regulation (155, 156), as summarized in **Figure 1**. A deficit in plasma serotonin has been reported in acute COVID, as well as in long COVID, compared to resolved COVID (205). However, two recent papers have strongly challenged the reality of this deficit (207, 208).

As shown in **Figure 1**, trp may be metabolized either in serotonin or in kynurenine (kyn). As indoleamine 2,3-dioxygenase (IDO) is a key enzyme for the degradation of trp in kyn, IDO activity is reflected in the trp/kyn (TKR) ratio. Kyn-derived kynurenic acid (KYNA) is an efficient anti-inflammatory molecule (see **Figure 1**). Upon inflammation, as IFN-γ can activate IDO (209), there is an immunoregulatory induction of IDO activity, and therefore a depletion of trp available for serotonin synthesis. A serum decrease in trp, an increase in kyn, or an decrease in the TKR ratio are hallmarks of inflammation (210). It is interesting to mention that IDO can also be induced by bacterial lipopolysaccharides (LPS) (211), considering the microbial translocation phenomenon characteristic of long COVID, ME/CFS, and possibly other PAIS. In long COVID and in ME/CFS, alterations of these biomarkers have all been reported (127, 200, 212, 213). Note that patients treated with IFN-α also show a strong decrease in the serum TKR ratio (214). Thus, an abnormally low plasma level of trp, as observed in long COVID and ME/CFS, could be viewed as an additional sign of persisting inflammation, with potential consequences on mental health.

### Influence of the gut-microbiota system in PAIS

The inflammatory state of the intestinal mucosa and of the enteric immune system has a considerable influence on the overall immune/inflammatory system (1, 105, 215), including on neuroinflammation (216). The intestine and its associated microbiota can produce anti-inflammatory molecules. In particular, short-chain fatty acids (SFCA), such as butyrate and propionate, are produced in the intestine by the fermentation of dietary fibers (217, 218), and can induce T_Regs_ (219, 220). The composition of gut microbiota, in particular the abundance of SFCA-producing bacteria, is altered in long COVID (221), in long Lyme (222), in ME/CFS (221, 223, 224) and in MS (225).

In different chronic inflammatory or viral diseases, a rupture of the integrity of the intestinal mucosal barrier is observed, affecting the tight junctions and allowing the translocation of commensal bacteria in the blood. This microbial translocation may also concern fungi (226), and be partly due to autoimmunity against components of the tight junctions (227). In post-acute viral syndromes, a loss of integrity of the gut mucosal barrier follows the virus-induced local inflammation (228–231). A loss of gut mucosa integrity has also been reported in ME/CFS patients (232, 233). In addition, whatever the initial infectious agent, an inflamed, damaged gut mucosa allows microbial translocation, accompanied by the release of the bacterial and inflammatory endotoxin LPS. This may create a self-sustained inflammatory phenomenon, accompanied and amplified by dysbiosis. It represents **fourth potential self-sustained inflammatory loop**. Such a microbial translocation could play an important role in autoimmune diseases (234), PAIS (228, 235, 236), as well as in HIV (229, 230).

Informative plasma markers of intestinal permeability are occludin and zonulin, which contribute respectively to the tight junction structure and to the regulation of their opening. Zonulin level rises sharply during severe acute COVID (236), a situation known to reflect a breakdown in the integrity of the intestinal barrier. In long COVID patients, plasma zonulin remains elevated, and fungal translocation from the gut to the blood can be evidenced (226), and elevated auto-antibodies against zonulin and occludin have been reported, as mentioned earlier (227). A clinical trial (NCT05747534) is currently being performed for evaluating the efficiency of lazarotide, an inhibitor of paracellular permeability, on children and young adults suffering from long COVID. Taken together, targeting the gut microbiota to reduce inflammation associated with PAIS appears as a relevant strategy, already proposed for MS (237), which is worth considering in future studies.

**Figure 2:**
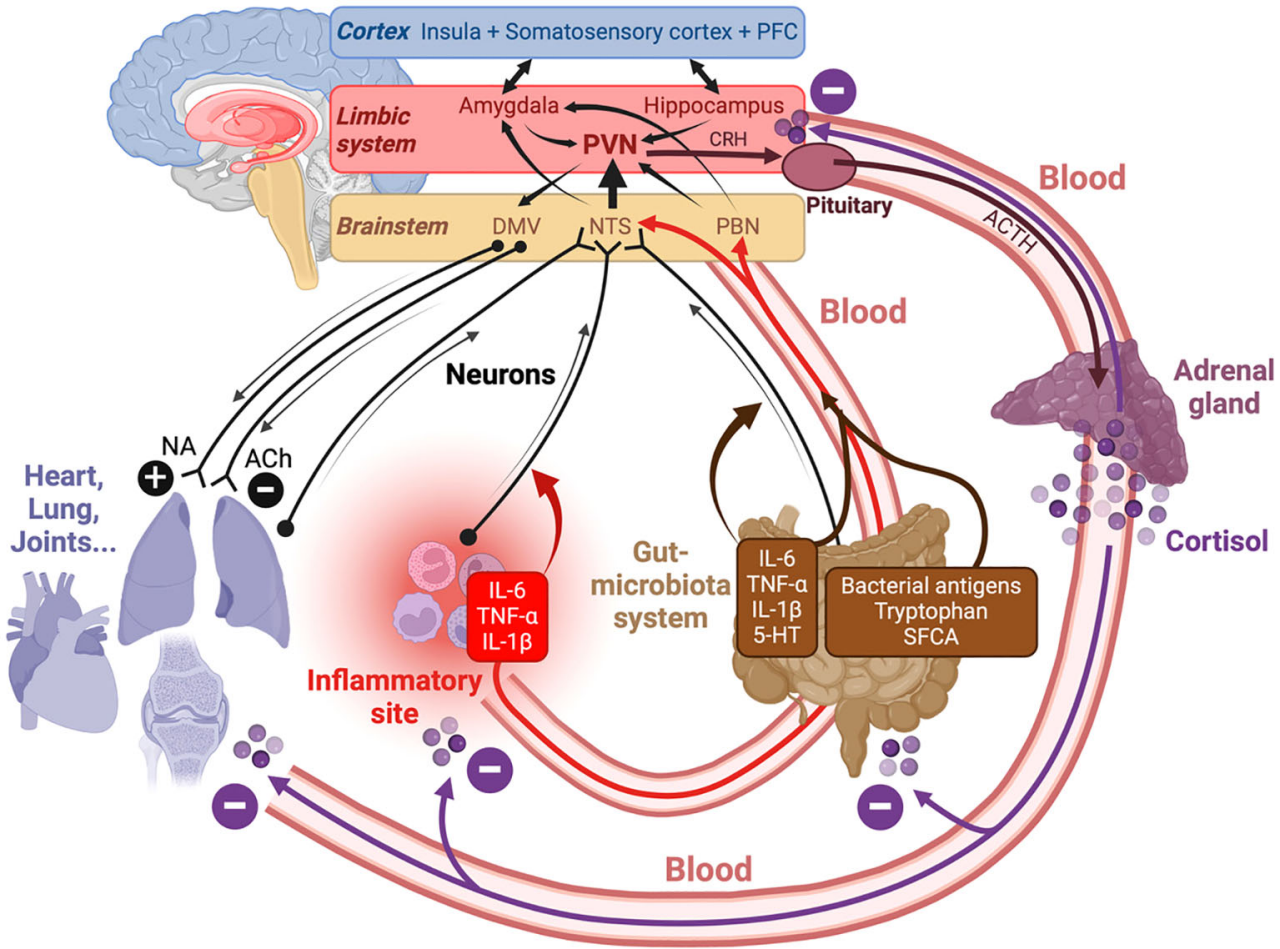
Bidirectional information exchange between the brain and the other organs is supported by neurons (black arrows) and blood (red arrows). Three parts of the brain (cortex, limbic system and brain stem) are involved in this dialog underlying interoception and homeostasis. Abbreviations: ACh, *acetylcholine*; ACTH, *adrenocorticotropic hormone;* DMV, *dorsal motor nucleus of the vagus*; NA, noradrenaline; NTS, *nucleus of the tractus solitarius;* PBN, *parabrachial nucleus;* PFC, *prefrontal cortex;* PVN, *paraventricular nucleus* of the hypothalamus. In the ANS, the afferent information converges on the NTS, whereas the efferent one is controlled by the DMV. The gut-microbiota system may produce both inflammatory (IL-6, TNF-α, IL-1β) an anti-inflammatory SFCA molecules, and the information sent to the brain via the blood and via the stimulation (e.g. by IL-6, IL-1β, TNF-α or 5-HT) of sensory fibers. Inflammatory cytokines can also be produced at inflammatory sites.

Concerning the microbiota-gut-brain (MGB) axis, the anti-inflammatory effects of SFCA produced by gut microbiota may affect multiple organs, including the lungs (238) and the brain (239–241). Other afferent blood-borne molecules on the MGB axis include bacterial antigens (242) and inflammatory cytokines such as IL-6, IL-1β and TNF-α, which may be produced in a context of gut inflammation. An additional mode of communication from the gut to the brain includes the enteric nervous system (ENS) and sensory neurons expressing receptors to TNF-α, IL-1β or IL-6 (243), and to 5-HT (244), which is abundantly produced at the gut level (245) (upwards black arrows in **Figure 2**). In the other direction, the brain may exert an influence on the gut, either via the autonomic and enteric nervous systems (downwards black arrows in **Figure 2**), or via activation of the Hypothalamus-Pituitary-Adrenal (HPA) axis (downwards red arrows in **Figure 2**), which will be examined in detail later. For instance, it has been shown that psychological stress leads to HPA-dependent monocyte-mediated exacerbation of gut inflammation (246, 247), and can alter the composition of the microbiota (248), a phenomenon possibly due to the presence of neurotransmitter receptors on bacteria (245).

In summary, given the major influence of the gut and its microbiota on the overall immune/inflammatory system and on neuroinflammation, and considering the importance of the MGB axis, one can foresee that well designed diets, complements or treatments aiming at reducing dysbiosis and gut inflammation may have a beneficial effect on different PAIS.

## Part II. Nervous system dependence of PAIS

Post-acute infection syndromes cannot be viewed only from a molecular/cellular point of view, nor from a purely psychic one. These two dimensions of PAIS must be taken into account, in order to have a chance of deciphering interactions between them. This key issue is absolutely required, but quite challenging, since these two dimensions correspond to traditionaly different epistemic fields.

### Interoception and the neuro-immune system

In what follows, we will see that in PAIS, the bidirectional exchange of information between the brain and the other organs is deeply perturbed, in other words interoception no longer works properly. A failed interoception is not specific of PAIS, but it plays a major role in PAIS. It is recalled that interoception is a process by which the brain senses, integrates and interprets signals originating from within the body, and sends feebacks aimed at maintaining homeostasis (249). It involves both the central and the autonomic nervous systems (CNS and ANS). ANS includes the sympathetic, parasympathetic and enteric nervous systems. Interoception involves brain regions belonging to three main levels: cerebral cortex, limbic system and brainstem (**Figure 2**). The main interoceptive cortical regions are the insula, the cingulate gyrus, the somatosensory cortex and some zones of the frontal cortex (250, 251). In the limbic system, the amygdala, the hippocampus and the hypothalamus are of particular importance for interoception. Interoceptive signals from the periphery converge on brainstem nuclei such as the NTS (*nucleus of the tractus solitarius*) and the PBN (*parabrachial nucleus*) (249). These brainstem nuclei are key for the detection of inflammatory stimuli, and project higher in the brain, namely on the PVN (*paraventricular nucleus*) of the hypothalamus (252) and on the amygdala (253). In the reverse, brain to body direction, the DMV (*dorsal motor nucleus of the vagus)* controls visceral motor and respiratory networks (254).

The interoceptive informations reaching the brainstem, limbic and cortical parts of the brain allow the homeostatic adjustment of body variables. Interoception has both an automatic, unconscious component (visceral adjustments, not requiring necessarily a cortical involvement) and a conscious one (e.g., behaviour changes). The fact that the brain is informed of a peripheral inflammation and can in turn act on this inflammation may be illustrated in many ways. First, there is the neuroendocrine HPA axis (133), examined in detail later. In addition, the ANS controls the vagus-mediated nervous inflammatory reflex (255). More recently, it has been shown that, still via the ANS, a peripheral inflammation can induce the appearance in the insular cortex, of inflammation-specific engrams associated with specific ensemble of neurons. A subsequent activation of these very same neurons can trigger a peripheral inflammation at the same location as the original one (256). More precisely, the physiological trace storing immune/inflammation-related information, the ‘‘immunengram,’’ is distributed between epigenetically modified neurons in the brain and immune memory cells residing in peripheral tissues (257).

This illustrates how the distinct features of the nervous and the immune systems complement each other to form an efficient neuro-immune system. Unlike the nervous system, with the brain as a central control structure, the immune system has no command post. On the other hand, the mobile cells of the immune system, which patrol the entire body, are capable of providing the immobile brain with decisive information on the state of all parts of the organism. This information is integrated by the brain which can then orient the functioning of the immune system. Thus, in order to apprehend the structural basis of interoception, one must take into account three brain levels: the cerebral cortex, limbic system and brainstem, and also consider the tight links between the nervous and immune systems, connected through slow blood circulation and fast neuronal activity.

### Dysautonomia, POTS and PEM

In PAIS, dysautonomia, i.e., a dysfunction of the ANS reveals a loss of equilibrium between its sympathetic, ergotropic and its parasympathetic, trophotropic components, with an impaired vagal, parasympathetic activity (258, 259). The PAIS-associated dysautonomia may affect multiple organs (lungs, heart, stomach, gut, kidney) and blood vessels. It may appear as an orthostatic intolerance, i.e., an inability to adapt hemodynamic parameters (cardiac frequency, blood pressure) to a vertical position, revealing a defective baroreflex-cardiovagal function. It may be unraveled either as Postural Orthostatic Tachycardia Syndrome (POTS), or as orthostatic-induced hypotension (260).

Exercise intolerance can even lead to Post-Exercise Malaise (PEM), a delayed and abnormal worsening of various symptoms and loss of energy following minimal physical or cognitive stressors or other triggers that would have been tolerated normally before disease onset (261, 262). POTS and PEM affect a fraction of patients suffering from ME/CFS or long COVID (6, 263, 264). PEM, also called Post-Effort Symptom Exacerbation (PESE) (265), can be best evidenced and provoked by two CardioPulmonary Exercise Test (CPET) 24 hours apart. In such a study with a cohort of ME/CFS patients compared to HC, a metabolomic analysis revealed in the ME/CFS patients, metabolic disruptions in lipid-related as well as energy-related pathways (266), reinforcing the relevance of the mitochondrial dysfunction hypothesis. A double CPET allows to distinguish clearly PEM/PESE from simple effort deconditioning (267).

Muscle abnormalities observed after induction of PEM (268) suggest that exercise may cause muscle injuries that do not heal normally in these patients. Note that the heat shock protein hsp70 is required for proper muscle healing (269). In post-infection ME/CFS patients, the plasma level of hsp70 was approximately 4 times lower than in HC (270). Moreover, after a maximal exercise, the hsp70 level shows a > 50% increase in HC, whereas a 15-30% decrease was observed in ME/CFS patients (270). This shows that **muscle repair after exercise may be compromised in ME/CFS patients**, and this could contribute to PEM.

In summary, dysautonomia reveals an inappropriate sympathetic/parasympathetic balance. It is reflected in Heart Rate Variability (HRV), which will be explained later. Dysautonomia may give rise to symptoms like POTS or PEM and may explain a large number of symptoms of PAIS such as hyperventilation, digestive, cutaneous and vascular, perpipheral neurological symptoms. In addition, PEM may be aggravated by problems in muscle repair. Clear manifestions of dysautonomia have been frequently reported for ME/CFS and long COVID, but much less so for long Lyme/PTLDS, for which further research on this topic would be welcome.

### The HPA axis and its relation with inflammation

As already mentioned, another major element of the brain-body dialog is the HPA axis (133, 271). A stress can trigger the release of Corticotropin-Releasing Hormone (CRH) by the hypothalamus, inducing the immediate production of AdrenoCorticoTropic Hormone (ACTH) by the pituitary, triggering in turn the release by the adrenal gland in the blood of adrenaline and cortisol. This response helps coordinating a series of physiological responses that range from an increase in heart rate to the suspension of digestion and of immune responses. It is anti-inflammatory and reduces pain sensitivity (272). Importantly, an elevation of cortisol in the brain circulation (in particular in the PVN and pituitary) results in an inhibition of HPA activation (**Figure 2**). The importance of such a negative feedback has been well described in the case of HPA axis dysfunctioning following traumatic brain injury (273), and thus contribute to a persistent dyshomeostatic state.

The stress can be mental or triggered by an infection/inflammation, an accident or a brain injury (133, 253, 273). Events triggered by these different types of causes can converge on the PVN of the hypothalamus. The PVN receives major inputs from the amygdala, which plays a key role in the management of emotional stimuli (253, 274), and from the PBN in the brainstem, a hub nucleus for the detection of inflammatory stimuli (252). Thus, the PVN is a major integrator of different types of stresses (**Figure 2**). Together with the inflammatory reflex of the ANS, (vagal, or cholinergic anti-inflammatory pathway) (255), the activation of the homologous HPA axis is crucial for preventing a potentially lethal cytokine storm, for instance in preventing severe acute COVID (275).

The stress-induced activation of ANS and endocrine outputs for a timely mobilization of energy resources are necessary for survival. However, exposure to traumatic or chronic stress can lead to autonomic imbalance, impaired negative feedback of the HPA axis, and illness (276). Thus, chronic stressful stimulations, whether of mental or physical origin, cause dysfunction of the HPA axis (133, 262). Chronic stimulation appears to gradually desensitize the HPA axis, impairing in particular its anti-inflammatory function, so that the body becomes unable to respond properly to new stressors. This is why chronic stimulation of the HPA axis is pro-inflammatory, whereas its acute stimulation is anti-inflammatory (277, 278). A model has been proposed (279) to explain the observation that prolonged stress makes the HPA less resilient to the next stress (280, 281).

When an infection or another stress is quickly resolved, the HPA axis returns to its normal functioning, which consists in being poised to be activated and deactivated quickly. Every day, cortisol levels show fluctuations whenever necessary to manage even low intensity events, such as waking up at the end of a night sleep, which may be viewed as a minor daily stress. The conspicuous blood cortisol awakening response is the net daily increase in blood cortisol within 1 hour of awakening (282). This cortisol arousal response is remarkably absent in patients suffering from a severe form of ME/CFS (20), from traumatic brain injury (273), from Post-Traumatic Stress Disease (PTSD) (283). The loss of this arousal response is a clear sign of HPA axis dysfunction, and an illustration of the fact that stress is poorly managed in PAIS. The question of basal blood cortisolaemia remains an unsolved question. In the current SARS-CoV-2 pandemic, hypocortisolaemia has been reported in one study (97), but not confirmed in other reports (284, 285). From our point of view, basal cortisol in the blood may not be a very relevant parameter, since its value changes all day long. The pertinent parameter should be the responsivity of the HPA axis network, as revealed by the magnitude of the cortisol awakening response, or the response to an evoked stress. For instance, it has been shown that after an experimental psychologic stress, the evoked cortisol response in fatigued breast cancer survivors is much smaller than that in nonfatigued ones, pointing to the involvement of the HPA axis in cancer-related fatigue (286).

For a better characterization of the relation between stress and the neuroendocrine system, the concept of allostatic load has been proposed. McEwen has defined allostatic load as the cost of chronic exposure to fluctuating or heightened neural or neuroendocrine response resulting from repeated or chronic environmental challenge that an individual reacts to as being particularly stressful (287). He has later described which cascade of events could explain the existence of a very interesting link between a psychic stress and a metabolic stress, including a mitochondrial one (288).

In summary, key aspects of body homeostasis, including the bidirectional functioning of the microbiota-gut-brain axis, rely on two parallel regulation systems, one purely nervous, the ANS, the other neuroendocrine, the HPA axis. A common hub for the two systems is the PVN of the hypothalamus, put into play following diverse perturbations like inflammation, and physical or psychic stress.

### Pathological and physiological fatigue

Fatigue is a major issue in PAIS. It is is caracteristized by a prolonged exhaustion state, uneasily relieved by rest and often exacerbated by physical or intellectual effort and emotions. Its precise origin and specificities (peripheral, central, objective or perceived) remain debated. To clarify this issue, let us first recall the difference between physiological and pathological fatigue. The physiological fatigue that anyone is familiar with is an internal state that prevents over-exertion and allows re-allocation and restoration of energetic resources. It is alleviated by rest and/or sleep. Contrary to a common misunderstanding, physiological fatigue is radically different from the pathological fatigue which can deeply alter the life of people suffering from PAIS, PTSD, cancer, neurodegenerative diseases, denutrition, aging or prolonged absence of physical activity. Pathological fatigue may be partially alleviated, but never eliminated, by physical, intellectual and emotional rest and/or sleep (non-restorative sleep).

Physiological fatigue corresponds to a homeostatic process, selected by evolution for its usefulness, whereas pathological fatigue unravels a dyshomeostatic state, which reflects a combined dysfunctioning of numerous potential systems, revealed by both physiological issues (chronic inflammation, mitochondrial dysfunctioning, cardio-pulmonary pathologies, endocrinopathies, vitamin deficiencies) and interoceptive processes, with inappropriate corrections by the brain of biological perturbations. What follows is an attempt to take into account these two roots (purely physiological and interoceptive) of pathological fatigue, and to try deciphering how they interact with each other.

There is no clear boundary between peripheral and central components of fatigue. For instance, in patients suffering from a PTSD like Gulf War Illness, with a key cerebral contribution, a mitochondria dysfunction has been detected in muscle biopsies, and the severity of the symptoms is correlated with that of the mitochondrial dysfunction (289). The efficiency of HyperBaric Oxygen Therapy (HBOT) in relieving symptoms of PTSD, in particular cognition, up to 2 years after the HBOT treatment (290), might be mediated by an effect on mitochondrial functioning (290). HBOT has also allowed an improvement of cognitive and psychiatric symptoms in long COVID patients (291, 292). However, publications on the usefulness of HBOT in PAIS remain scarce, several clinical trails are on going and HBOT information concerning potential toxicities and intolerance still need to be improved.

At the brain level, fatigue arising from interoceptive networks involves feelings of tiredness, lack of energy, and difficulty in concentrating. To understand the origins of perceived fatigue, it is important to take into account different kinds of potential cellular stresses in periphery, and the information sent to the brain (via the blood circulation, the vagus nerve and sensory inputs). This information may then give rise on one hand to an interoceptive, anti-inflammatory feedback signal sent to the inflamed region via ANS-derived catecholamines and HPA axis-derived corticosteroids, and on the other hand, to the involvement of limbic and cortical brain regions, which modulate the interoceptive networks by involving emotion, memory and cognition.

Given the permanent bidirectional communication between the brain and the other parts of the body in interoceptive networks, it would not be realistic to aim at fully disentangling the central and peripheral components of fatigue. People suffering from MS who report elevated levels of fatigue exhibit alterations in interoceptive networks functioning (particularly in the insula and dorsal anterior cingulate cortex) (293). It is in the brain that fatigue is perceived, but the location of the interoceptive sensors signalling deviations from a balanced homeostatic state remains unclear. Muscular fatigue and fatigue perception are both strongly influenced by oxidative stress and inflammation (294). Interoceptive signals may reach the brain in many ways. As shown in **Figure 2**, they may first activate vagus nerve terminals which are located in the gut and project on the NTS in the brainstem. The NTS sends outputs higher in the brain, e.g. to the PVN of the hypothalamus and the amygdala (253). In the reverse direction, CNS-derived cytokines may induce muscular weakness. Indeed, infection and chronic disease activate a systemic brain-muscle signaling axis in which CNS-derived cytokines directly regulate muscle physiology, causing muscle mitochondrial dysfunction and impaired motor function (295). Thus, there are both central and peripheral sensors related to perceived fatigue, and a complex bidirectional signalling between muscles and brain has to be taken into account.

### Nociception, inflammation and depression associated with PAIS

We have seen above that the brain responses to stress and to inflammation involve common structural and functional elements. This neuro-immune connection is reinforced by the nociceptive system, which plays two distinct roles: not only does it may inform the brain of a peripheral disorder, but it is also plays an active role in peripheral inflammation (296). Nociceptive neurons are endowed with a series of receptors, such as innate immune system receptors, like Toll-like receptors (TLR), which can directly detect the presence of a pathogen and send danger signals to the immune system, for instance through the secretion of neuropeptides that activate immune cells. Indeed, many neurotransmitter receptors are expressed by immune cells (297). Neurons and immune cells share the same molecular vocabulary and language, which constitutes a basis for the strong connexions that exist between inflammation, stress, and pain.

There are tight links between inflammation and depression/anxiety. Evidence for these links are multiple, as summarized by (253): 1) Injecting mice with LPS or inflammatory cytokines induces a depression-like behavior. 2) Inflammatory markers such as IL-1β, IL-6, or CRP are on average higher in patients suffering from severe depression. 3) The frequency of depression is higher in patients suffering from chronic inflammatory pathologies such as MS. 4) Patients treated with IFN-α suffer from significant side effects including severe fatigue, anxiety, and depression. The neurotoxicity of IFN-α is well established (298), as well as the cognitive impairment induced by this cytokine (299). 5) Some medications like fluoxetine (an inhibitor of 5-HT capture) have both anti-inflammatory and anti-depressant effects (205, 300). An anti-TNF-α antibody not only has an anti-inflammatory effect but can also alleviate fatigue in patients suffering from rheumatoid arthritis (301).

### Questionnaires and functional tests

How could the wide range of physiopathological phenomena underlying PAIS symptoms be taken into account to help designing a PAIS diagnosis tool? We have seen that different perturbations associated with PAIS can be associated with different biomarkers, which however reveal insufficient for an optimized PAIS diagnosis. To improve this diagnosis, it is advisable to rely on questionnaires and functional tests, in addition to biomarker measurements. A Quality of Life questionnaire like SF-36 covers many health parameters (302) clustered along 8 axes, including physical functioning, social functioning, general health, vitality/energy/fatigue. An unsupervised analysis of SF-36 data can lead to a 2D representation based on principal component analysis, which has allowed a fully non ambiguous distinction of ME/CFS patients and of HC (303). For longitudinal studies requiring repeated questionnaires, the shorter EQ-5D-5L Quality of Life questionnaire may be more relevant (304). A large set of questionnaires may be used for assessing specifically fatigue, anxiety, depression, quality of sleep, autonomic dysfunction (see e.g (127, 305).

Clinical testing for POTS can easily be performed with an office-based test, the 10-min NASA Lean Test (NLT), which only requires a pulse oximeter and a blood pressure cuff. This test has recently been described in detail and applied to patients suffering from either long COVID or ME/CFS (306). This study has allowed to highlight the link between orthostatic intolerance and cognitive impairment in long COVID and a subset of ME/CFS patients, a phenomenon that may be associated to both fatigue and dysautonomia. In the NLT, cognitive testing is done with a smartphone application, the Defense Automated Neurobehavioral Assessment (DANA) Brain Vital application. The 5-minute DANA Brain Vital test suffices to quantify different reaction time measurements and sustained attention.

One feature of dysautonomia associated with PAIS, the inability to sustain an intense effort, can be measured during a CPET (cardiopulmonary exercise test). In a CPET, one can determine *VO*_2max_, i.e., the peak oxygen consumption during a maximum effort, an objective measure of an individual maximum energy producing capacity. In long COVID patients, this VO_2max_ is 30-45% lower than that in HC (268, 307). It is also abnormally low in ME/CFS patients (308, 309). Note that a CPET may be at risk of inducing a PEM, i.e., after a CPET, the patient may suffer for days or even weeks of an exacerbation of the symptoms. Inducing on purpose a PEM (266, 310) with potentially severe consequences is ethically questionable unless appropriate precautions are taken, which is feasible (311). PEM may also be revealed by the DePaul Symptom Questionnaire (312).

Another measurable parameter is heart rate reserve, i.e., the difference between the resting heart rate and the rate during a maximal effort. Again, heart rate reserve is significantly smaller in ME/CFS patients than in HC (309, 313). CPET allows also to measure HRV (heart rate variability), which reflects how adaptable the body is to sudden changes (in response to a stress or simply to a position change). It is important to mention that HRV is a biomarker that can be recorded and analyzed independently without needing another study, such as CPET. It is a non-invasive method widely used in physiological and pathophysiological conditions. It allows for the assessment of the body’s physical and mental health. A highly variable heart rate indicates a good equilibrium between the sympathetic and parasympathetic influences, so that the body can adapt to many kinds of changes, whereas a low HRV (usually resulting from a deficient parasympathetic contribution) implies a deficient stress adaptability. Compared to that in HC, HRV is significantly smaller in ME/CFS (314) and in long COVID patients (315). Thus, a low HRV may be a clear sign of dysautonomia. A large number of studies (see e.g (316).) make use of HRV measurements in the frame of the polyvagal theory of Stephen Porges (317). This theory “speculates that mammalian, but not reptilian, brainstem organization is characterized by a ventral vagal complex related to processes associated with attention, motion, emotion, and communication”. However, this central assumption has been shown to be a major oversimplification (318). Within the frame of the polyvagal theory, HRV is used not only as a proxy of the general activity of the ANS, but it can also mirror the emotion-cognition interactions (319). This again is a major oversimplification, as the theory (and the use of HRV as a general proxy) assumes namely that consistent patterns in the activity of different vegetative efferents (towards the heart, lung, gut etc…) should be observed, but this prediction is frequently not verified (319). Because HRV corresponds to a low cost, non-invasive, easy to record measurement, it has been used in thousands of publications, which do not necessarily refer to the polyvagal theory (320).

## Conclusion

The difficulties in apprehending, diagnosing, and treating chronic diseases like PAIS are first due to their multidimensionality. Decades of reductionism and of hyper-specialization in scientific research have not prepared most scientists and physicians to integrate data from quite distinct epistemic fields. One cannot have a global view of PAIS without taking into account phenomenons as diverse as fatigue, hypometabolism, mitochondrial dysfunction, inflammation, dysbiosis, autoimmunity, dysautonomia, orthostatic intolerance, PEM, or poor stress coping, among other diverse and multidimensional concepts.

**Figure 3:**
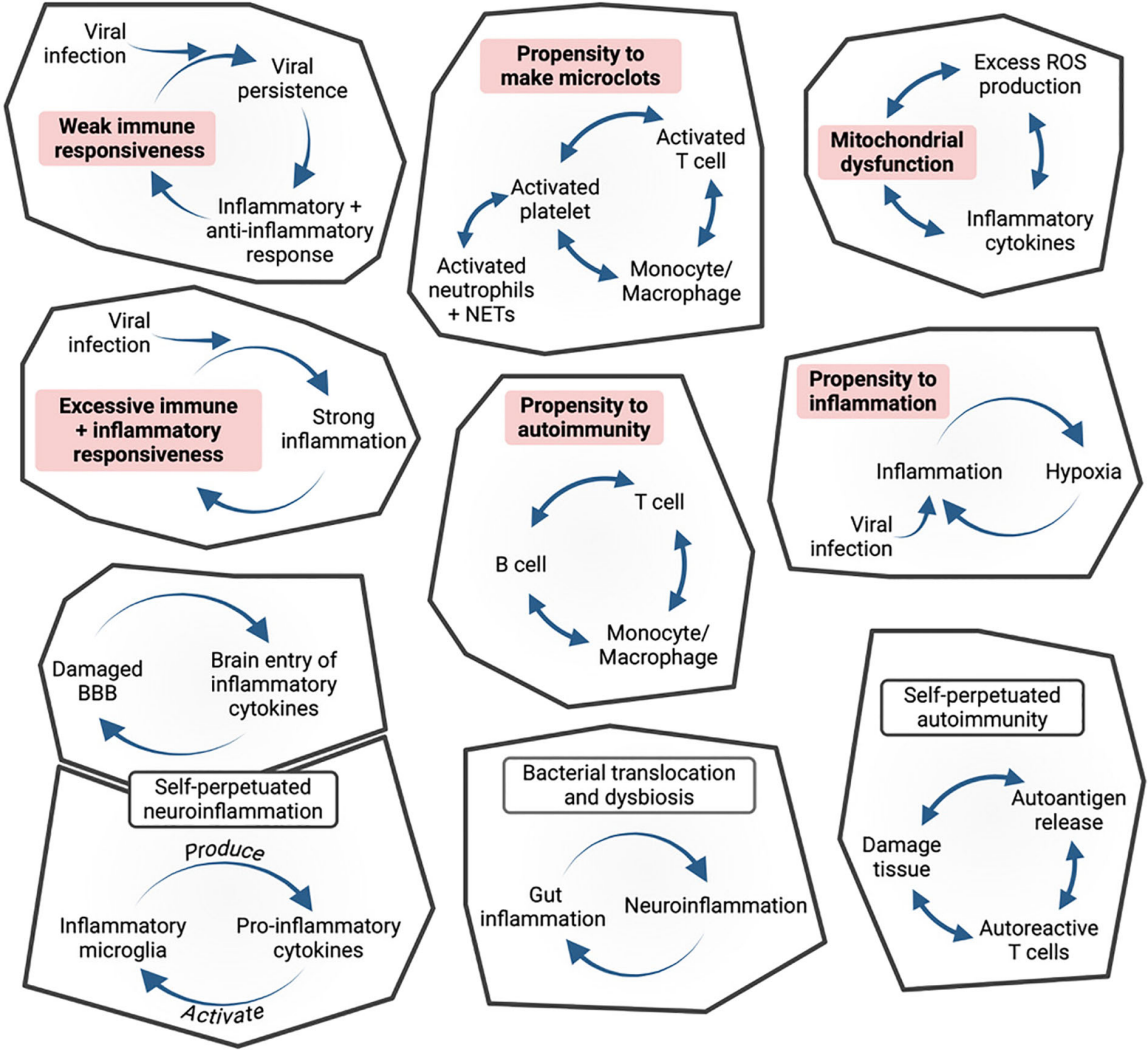
Diverse individual predispositions (bold on pink background), after an infection, may lead to a whole series of functional vicious circles. These predispositions- and infection-induced self-sustained circles constitute the building blocks of PAIS.

A second problem for apprehending PAIS is that, whatever the initial etiology, chronicity implies mechanisms of self-perpetuation, not in a steady way, but involving in most cases flares and remissions. Chronicity may involve a large set of potential mechanisms, all rooted in individual predispositions. **Figure 3** illustrates a series of examples of vicious circles potentially triggered by a poorly resolved infection, depending on such predispositions. I have mentioned earlier **four types of potential self-sustained inflammatory loops**, one formed by the T cell, B cell and monocyte triangle, a second one due to the fact that microglial cells can both produce and be activated by inflammatory cytokines, a third one due to excessive unbuffered ROS levels and damaged mitochondria, leading to less ATP and more ROS production, and a fourth one to bacterial translocation through a damaged gut mucosa, which may sustain mucosal inflammation and permeability. In addition, the microglial-gut-brain axis may be dysfunctional in a bidirectional way (245). Moreover, an inefficient immune system may favour viral persistence (321), or reactivation of latent viruses, whereas an overreactive immune system may lead to an excessive inflammation (89). Self-sustained interactions between platelets, neutrophils, T cells and monocytes/macrophages can favour the formation of microclots (141). In a context of painful inflammation, nociceptors not only transmit an information to the brain, but can amplify inflammation (296, 322). Thus, in addition fo pathogen persistence, a number of phenomena may contribute to theperpetuation of PAIS.

A central problem for apprehending PAIS is related to the variety of individual frailties (or risk factors) that may predispose to develop a PAIS. In addition to the genetic background (e.g. related to mast cells or mitochondria), a number of key factors depend on environment and individual history, i.e., lifestyle, toxins, past infections that may leave an *immunological scar* (1), with elements that are memorized through epigenetics marking of hematopoietic stem cells (3) or in immunengrams of the neuro-immune system (257). Simple chance *bystander activation* of pre-existing auto-reactive T cells may also play a role in infection-induced auto-immunity.

**Figure 4:**
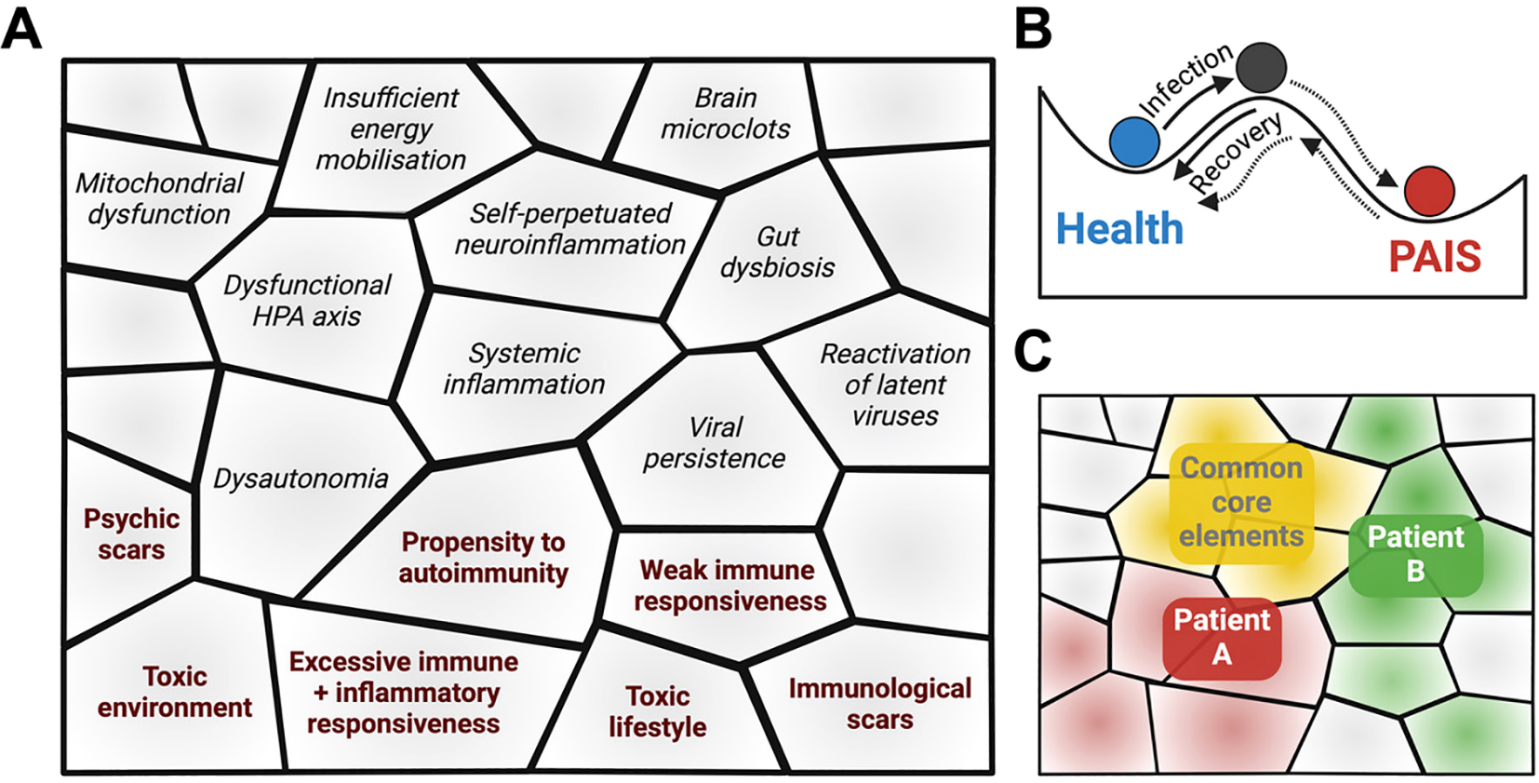
**(A)** PAIS building blocks may self-assemble to create a metaphoric pavement. Examples of individual frailties preexisting the disease are marked in bold and pink. The other building blocks are examples of infection-induced symptoms. **(B)** This pavement forms the PAIS basin of attraction, distinct form the Health basin of attraction. Basins of attraction are used in the sense used by Waddington to define an epigenetic landscape. Black and gray lines correspond to high and low probabilities of going from one point of the epigenetic landscape to another one. **(C)** The self-assembled pavements of two PAIS patients (red and green) may include both common core elements (yellow) and diverse ones.

**Figure 3** illustrates how each one of these predispositions and of self-sustained circles may constitute building blocks that can self-assemble in many possible ways (just like molecules can self-assemble), to give rise to PAIS. These building blocks can be viewed as forming a pavement (**Figure 4A**), at the bottom of a basin of attraction, in the sense given by Conrad Waddington when describing what he called the *epigenetic landscape* (323, 324). In this figure, examples of individual frailties preexisting the disease are marked in bold on a gray background. As depicted in **Figure 4B**, health and PAIS could constitute two basins of attraction, the transition form health to PAIS being triggered by an infection. The interactions between different PAIS building blocks strengthen the formation of a deep PAIS basin of attraction, i.e., a stabilized dyshomeostatic state (204, 325, 326). In particular, core symptoms (extenuating fatigue, cognitive problems, sleep disorders and pain) not only strengthen but often exacerbate each other, leading to isolation, depression, and sometimes even to a state that looks like PTSD.

A given PAIS, e.g., long COVID, could be viewed as being itself a group of several diseases, with different possible pavements, i.e., different self-assemblies of building blocks, at the bottom of PAIS basins of attraction, and different depths of these basins. **Figure 4C** shows that different basins of attraction may be paved by common core building blocks and by variable ones. For each patient, an optimal diagnosis would unravel the links between the disease and his/her specific initial problematic terrain, which in most cases remains uncharacterized. I propose that targeting a single symptom/component of the disease, i.e., a single building block of the PAIS basin of attraction is unlikely to modify its depth enough to give a chance of escaping this attractor. The simultaneous targeting of several building blocks of a given patient appears necessary to make a recovery possible. Admittedly, it is much easier to design clinical trials with a single potential treatment than with a combination of treatments, but efforts should be made in the direction of treatments that would be combined on sound arguments.

Efficient clinical trials will require the prior characterization of objective clusters of patients corresponding to different subtypes of the disease. To this end, a key issue concerns the choice of discriminating clustering factors. While proteomics allows the identification of inflammatory and non-inflammatory clusters, their overlap is such that these clusters are not of great help for an individual diagnosis (129, 131, 327). A better clusterisation has been obtained for long COVID, based on differentially methylated CpGs, i.e., on an epigenetic signature in PBMCs (328), which, not surprisingly, is at the basis of modifications of gene expression by individual histories.

An even better discrimination of PAIS subtypes should be obtained with a composite profiling taking into account PAIS multidimensionality. The elements to consider in such a composite profiling, for a principal component analysis, could be for instance the following. 1) A set of well identified inflammatory biomarkers (IL-6, TNF-α, IL-1β, IFN-α, neutrophil and NET biomarkers) 2) Additional biomarkers including the awakening cortisol response, zonulin, tryptophan, kynurenine 3) Key quantified questionnaire-based symptoms (fatigue, ability to concentrate and to memorize) and number of symptoms 4) Functional tests including HRV, VO_2max_, orthostatic intolerance and cognitive tests.

Such a new composite profiling is expected, following principal component analyses and the use of AI to make the best use of a large set of data in a multidimensional space, to provide a useful subtyping of PAIS, opening the door to future individual treatments based on a precision diagnosis.

## Footnotes

1
https://www.mypharma-editions.com/geneuro-annonce-les-resultats-de-lessai-gnc-501-dans-le-syndrome-post-COVID-19.
